# Supplementary material for: Analysis of PPARγ Signaling Activity in Psoriasis
Source: Int J Mol Sci. 2021 Aug 10;22(16):8603. doi: 10.3390/ijms22168603 (PMC8395241; doi:10.3390/ijms22168603)
Supplement: Supplementary file 1 [file ijms-22-08603-s001.zip › Supplemental materials_Analysis of PPARg signaling activity in psoriasis/Pathway models/Models images and html files/Differentiation of psoriatic T cells/10999.html]

STAT4


# Protein STAT4

|  |  |
| --- | --- |
| URN | urn:agi-llid:6775 |
| Total Entities | 0 |
| Connectivity | 1224 |
| Name | STAT4 |
| Description | signal transducer and activator of transcription 4 |
| Notes | The protein encoded by this gene is a member of the STAT family of transcription factors. In response to cytokines and growth factors, STAT family members are phosphorylated by the receptor associated kinases, and then form homo- or heterodimers that translocate to the cell nucleus where they act as transcription activators. This protein is essential for mediating responses to IL12 in lymphocytes, and regulating the differentiation of T helper cells. Mutations in this gene may be associated with systemic lupus erythematosus and rheumatoid arthritis. Alternate splicing results in multiple transcript variants that encode the same protein. [provided by RefSeq, Aug 2011] |
| Primary Cell Localization | Nucleus |
| Class | Transcription factor |

---

|  |  |
| --- | --- |
| Pathway | CD8+ T-Cell Activation |
|  | Th1-Cell Differentiation |
|  | Th1-Cells Activation and Proliferation in Atherosclerosis |
|  | Lymphocyte Mediated Myocardial Injury in Myocarditis |
|  | Proteins Involved in Myocarditis |
|  | Atopic Dermatitis |
|  | T-Cells Differentiation Block in Psoriasis |
|  | Th1-Cell Function in Systemic Lupus Erythematosus |
|  | T-Cell Cytotoxic Response against Melanocytes in Vitiligo |
|  | Tubular Cell Dysfunction in Progressive Diabetic Nephropathy |
|  | CD4+T-Cell Response in Celiac Disease |
|  | Th1-Cell Activation in Crohn's Disease |
|  | Proteins Involved in Ulcerative Colitis |
|  | Proteins Involved in Rheumatoid Arthritis |
|  | IL12 Antitumor Effect is Blocked in Cancer |
|  | IL27R -> STAT Signaling |
|  | IL12R -> STAT Signaling |
|  | UrokinaseR -> STAT Signaling |
|  | NOTCH -> Expression Targets in Thymus |
|  | Elevated Receptors -> Expression Targets in Testis |
|  | PPAR Psoriasis |
|  | Model of PPARG signaling in psoriasis |
|  | New Pathway (3) |
|  | Figure 3\_Mixed network of dexamethasone targets |
|  | Figure 3\_Mixed network of dexamethasone targets |
|  | 1\_Differentiation of psoriatic T cells |
|  | 1\_Defects in response to pathogens in the gut promotes inflammation in Crohn's disease |
|  | 4\_1\_Polymorphisms associated with inflammatory bowel diseases |
|  | Differentiation of psoriatic T cells |

---

|  |  |
| --- | --- |
| Group | Genes with Mutations Associated with Psoriasis |
|  | Genes Associated with Systemic Lupus Erythematosus |
|  | Genes with Mutations Assosiated with Rheumatoid Arthritis |

---

|  |  |
| --- | --- |
| MedScan ID | 6775 |

---

|  |  |
| --- | --- |
| LocusLink ID | 6775 |
|  | 20849 |
|  | 367264 |

---

|  |  |
| --- | --- |
| Alias | signal transducer and activator of transcription 4 |
|  | transcription factor STAT4 |
|  | STAT4 |
|  | signal transducers and activators of transcription 4 |
|  | OTTHUMP00000163553 |
|  | SLEB11 |
|  | STAT4 transcription factor |
|  | STA4\_MOUSE |
|  | signal transducer and activator of transcription 4 variant 3 |
|  | Systemic lupus erythematosus, susceptibility to, 11 |
|  | signal transducer and activator of transcription IV |

---

|  |  |
| --- | --- |
| GO ID | 0001228 |
|  | 0003700 |
|  | 0000981 |
|  | 0000978 |
|  | 0007259 |
|  | 0019221 |
|  | 0006952 |
|  | 0035722 |
|  | 0038114 |
|  | 0038155 |
|  | 0070757 |
|  | 0045944 |
|  | 0042127 |
|  | 0043434 |
|  | 0005829 |
|  | 0005654 |
|  | 0005634 |
|  | 0003677 |
|  | 0043565 |
|  | 0008283 |
|  | 0071310 |
|  | 0010628 |
|  | 0006468 |
|  | 0006355 |
|  | 0007165 |
|  | 0005737 |
|  | 0071345 |
|  | 0071407 |
|  | 0061326 |
|  | 0006351 |
|  | 0000977 |
|  | 0004871 |
|  | 0000979 |
|  | 0042803 |
|  | 0000983 |
|  | 0005164 |
|  | 0035458 |
|  | 0060333 |
|  | 0046725 |
|  | 0043124 |
|  | 0016525 |
|  | 0001937 |
|  | 0033209 |
|  | 0000790 |
|  | 0005730 |
|  | 0048471 |
|  | 0005509 |
|  | 0003690 |
|  | 0019899 |
|  | 0031663 |
|  | 0043330 |
|  | 0009617 |
|  | 0032496 |

---

|  |  |
| --- | --- |
| KEGG ID | hsa:6775 |
|  | mmu:20849 |
|  | rno:367264 |

---

|  |  |
| --- | --- |
| Organism | Homo sapiens {Organism urn:agi-taxid:9606} |
|  | Mus musculus {Organism urn:agi-taxid:10090} |
|  | Rattus norvegicus {Organism urn:agi-taxid:10116} |
|  | Mus musculus |
|  | Homo sapiens |
|  | Rattus norvegicus |

---

|  |  |
| --- | --- |
| Mouse chromosome position | 1 26.67 cM |
|  | 1 25.9 cM |

---

|  |  |
| --- | --- |
| OMIM ID | 600558 |
|  | 612253 |
|  | 180300 |
|  | 152700 |

---

|  |  |
| --- | --- |
| Rat chromosome position | 9q22 |

---

|  |  |
| --- | --- |
| Hugo ID | 11365 |
|  | HGNC:11365 |

---

|  |  |
| --- | --- |
| Human chromosome position | 2q32.2-q32.3 |

---

|  |  |
| --- | --- |
| Swiss-Prot Accession | Q14765 |
|  | Q14765.1 |
|  | P42228 |
|  | Q3V157 |
|  | P42228.1 |
|  | Q66HB2 |
|  | Q96NZ6 |
|  | Q53RU2 |
|  | Q53S87 |
|  | Q59GR6 |
|  | Q3U098 |
|  | Q4G0C1 |
|  | Q8C2A4 |
|  | O70428 |

---

|  |  |
| --- | --- |
| PIR ID | A56047 |

---

|  |  |
| --- | --- |
| GenBank ID | NC\_000002 |
|  | XM\_011511705 |
|  | XP\_011510007 |
|  | NM\_001243835 |
|  | NP\_001230764 |
|  | NM\_003151 |
|  | NP\_003142 |
|  | XM\_006712719 |
|  | XP\_006712782 |
|  | XM\_017004784 |
|  | XP\_016860273 |
|  | NG\_012852 |
|  | AB111353 |
|  | AC067945 |
|  | AAY24184 |
|  | AC079777 |
|  | AAY15055 |
|  | AF423072 |
|  | AAL12164 |
|  | AX354560 |
|  | CAD22977 |
|  | CH471058 |
|  | EAX10846 |
|  | EAX10847 |
|  | EAX10848 |
|  | EAX10849 |
|  | DQ003482 |
|  | KT584956 |
|  | AB209043 |
|  | BAD92280 |
|  | AK299976 |
|  | BAG61799 |
|  | AK300868 |
|  | BAG62516 |
|  | AK301172 |
|  | BAG62759 |
|  | BC016694 |
|  | BC031212 |
|  | AAH31212 |
|  | BF966132 |
|  | DC306142 |
|  | DC399230 |
|  | EU304788 |
|  | ACA01857 |
|  | EU304789 |
|  | ACA01858 |
|  | EU304790 |
|  | ACA01859 |
|  | EU304791 |
|  | ACA01860 |
|  | EU304792 |
|  | ACA01861 |
|  | EU304793 |
|  | ACA01862 |
|  | L78440 |
|  | AAB05605 |
|  | MF781077 |
|  | ATE87180 |
|  | Q14765 |
|  | NC\_000067 |
|  | XM\_006495815 |
|  | XP\_006495878 |
|  | XM\_006495814 |
|  | XP\_006495877 |
|  | NM\_001308266 |
|  | NP\_001295195 |
|  | XM\_006495816 |
|  | XP\_006495879 |
|  | NM\_011487 |
|  | NP\_035617 |
|  | AC108840 |
|  | AC123752 |
|  | CH466548 |
|  | EDK99962 |
|  | EDK99963 |
|  | AK037598 |
|  | AK088991 |
|  | BAC40690 |
|  | AK132675 |
|  | BAE21295 |
|  | AK157083 |
|  | BAE33957 |
|  | BC052907 |
|  | BC098499 |
|  | AAH98499 |
|  | BF020635 |
|  | BY097735 |
|  | BY750146 |
|  | U06923 |
|  | AAA19453 |
|  | U09351 |
|  | AAA19692 |
|  | P42228 |
|  | NC\_005108 |
|  | NM\_001012226 |
|  | NP\_001012226 |
|  | AC\_000077 |
|  | AABR07067608 |
|  | AABR07067609 |
|  | AABR07067610 |
|  | AAHX01059849 |
|  | AAHX01059850 |
|  | AF055291 |
|  | AAC12758 |
|  | BC081938 |
|  | AAH81938 |
|  | XM\_011511704 |
|  | XP\_011510006 |
|  | XM\_005246817 |
|  | XP\_005246874 |
|  | XM\_011511706 |
|  | XP\_011510008 |
|  | NC\_018913 |
|  | AC092614 |
|  | AMYH02005442 |
|  | AMYH02005443 |
|  | HG495061 |
|  | DQ891548 |
|  | ABM82474 |
|  | DQ894736 |
|  | ABM85662 |
|  | AC\_000023 |
|  | AAHY01006299 |
|  | AAHY01006300 |
|  | AAHY01006301 |
|  | AAHY01006302 |
|  | AAHY01006303 |
|  | AAHY01006304 |
|  | AAHY01006305 |
|  | AAHY01006306 |
|  | AC\_000134 |
|  | ABBA01062392 |
|  | ABBA01062393 |
|  | ABBA01062394 |
|  | ABBA01062395 |
|  | NT\_005403 |
|  | NW\_921585 |
|  | NW\_001838863 |
|  | AC\_000045 |
|  | Q53RU2 |
|  | Q53S87 |
|  | Q59GR6 |
|  | NT\_039170 |
|  | NW\_001030680 |
|  | Q3U098 |
|  | Q3V157 |
|  | Q4G0C1 |
|  | Q8C2A4 |
|  | NW\_047815 |
|  | NW\_001084882 |
|  | O70428 |
|  | Q66HB2 |

---

|  |  |
| --- | --- |
| Swiss-Prot ID | STAT4\_HUMAN |
|  | STAT4\_MOUSE |
|  | Q66HB2\_RAT |
|  | Q3V157\_MOUSE |

---

|  |  |
| --- | --- |
| Cell Localization | Cytoplasm |
|  | Nucleus |

---

|  |  |
| --- | --- |
| Ensembl ID | ENSG00000138378 |
|  | ENSP00000351255.4 |
|  | ENST00000358470.8 |
|  | ENSP00000376134.2 |
|  | ENST00000392320.7 |
|  | ENSMUSG00000062939 |
|  | ENSMUSP00000130713.1 |
|  | ENSMUST00000168302.7 |
|  | ENSMUSP00000027277.5 |
|  | ENSMUST00000027277.6 |
|  | ENSRNOG00000014079 |
|  | ENSRNOP00000020032.5 |
|  | ENSRNOT00000020032.6 |
|  | ENST00000392320.6 |
|  | ENSP00000351255 |
|  | ENST00000358470 |
|  | ENSP00000376134 |
|  | ENST00000392320 |
|  | ENSMUSP00000027277 |
|  | ENSMUST00000027277 |
|  | ENSRNOP00000020032 |
|  | ENSRNOT00000020032 |
|  | ENSMUSP00000130713 |
|  | ENSMUST00000168302 |
|  | ENSRNOG00000050942 |

---

|  |  |
| --- | --- |
| MGI ID | MGI:103062 |
|  | 103062 |

---

|  |  |
| --- | --- |
| RGD ID | 1305747 |

---

|  |  |
| --- | --- |
| Unigene ID | Hs.80642 |
|  | Mm.1550 |
|  | Rn.137580 |
|  | Hs.735572 |

---

|  |  |
| --- | --- |
| Homologene ID | 20679 |

---

|  |  |
| --- | --- |
| Shape | O-vertex |

---

|  |  |
| --- | --- |
| IPI ID | IPI00114970 |
|  | IPI00000106 |
|  | IPI00471606 |
|  | IPI00649102 |

---
